# Immune Response to Bioluminescence Imaging Reporters in Murine Tumor Models

**DOI:** 10.1007/s11307-025-02010-7

**Published:** 2025-04-15

**Authors:** Angisha Basnet, Dylan D. Thomas, Kaitlyn M. Landreth, F. Heath Damron, Tracy W. Liu

**Affiliations:** 1 Department of Microbiology, Immunology, and Cell Biology, West Virginia University, Morgantown, WV 26506 USA; 2 Vaccine Development Center, West Virginia University Health Sciences Center, Morgantown, WV 26506 USA; 3 WVU Cancer Institute, West Virginia University, Morgantown, WV 26506 USA

**Keywords:** Bioluminescence imaging, Luciferase, Tumor microenvironment, Immunology

## Abstract

**Purpose:**

Imaging reporters have been widely employed in cancer research to monitor real-time tumor burden and metastatic spread. These tools offer a valuable approach for non-invasive imaging of tumor dynamics over time. With the established understanding that tumor immunology plays a critical role in cancer progression, it is essential to ensure that the chosen imaging reporters used to study tumor-immune interactions do not inadvertently elicit an immune response. This study aimed to investigate the immune response to bioluminescence reporters used for *in vivo* tracking of tumor cells in immunocompetent murine models.

**Procedures:**

The *in vitro* and *in vivo* growth effects of two stably expressed bioluminescence reporter genes, a red-shifted firefly luciferase and a click beetle green luciferase, were evaluated in four different cancer cell lines. Differences in parental and reporter-expressing cancer cell immune cell composition, activation, and secreted cytokine levels were evaluated using flow cytometry, cytokine arrays and ELISAs.

**Results:**

The data revealed no significant differences in *in vitro* cell proliferation between parental and reporter cancer cell lines. *In vivo* subcutaneous tumor growth was not observed in tumor cells stably expressing the red-shifted firefly luciferase. Cells labeled with click beetle green luciferase demonstrated no significant differences in *in vivo* subcutaneous tumor growth compared to parental cells. Tumor cells expressing red-shifted firefly luciferase induced an increase in activated and cytotoxic T cells compared to parental and click beetle green luciferase, suggesting enhanced immunogenicity. Furthermore, the tumor-immune composition and cytokine production were similar between parental and click beetle green luciferase-labeled tumor cells.

**Conclusions:**

These findings demonstrate that the stable expression of click beetle green luciferase in cancer cells, in contrast to red-shifted firefly luciferase, has minimal immunogenicity and does not alter tumor development in immunocompetent mice. We report detailed characterization studies of bioluminescence reporter cells, providing essential considerations for their use in investigating tumor-immune interactions in syngeneic murine tumor models.

**Supplementary Information:**

The online version contains supplementary material available at 10.1007/s11307-025-02010-7.

## Introduction

Real-time imaging with optical imaging reporters has advanced cancer research by facilitating the non-invasive study of tumor biology, including gene expression, cell proliferation, and metastasis [1–5]. Fluorescence reporters, first developed in the 1960s with the discovery of green fluorescent protein (GFP) from the jellyfish *Aequorea victoria*, revolutionized cancer research by enabling fluorescence imaging [6, 7]. Fluorescence imaging relies on the excitation of a fluorophore by an external light source, causing it to absorb light at a specific wavelength and emit a detectable photon at a longer wavelength [8]. In contrast, bioluminescence imaging, using a luciferase reporter, relies on the production of light through a biochemical reaction, involving the substrate luciferin, ATP, and oxygen [2, 9]. The first reference of bioluminescence was traced back to the Greek philosopher Aristotle in 384–322 BCE [10]. It was not until 1885 that Raphael Dubois discovered that the bioluminescence reaction in click beetles required two key components, naming the substrate luciferin and the enzyme luciferase [10]. Bioluminescence was first used for live *in vivo* imaging in 1995, where bacterial pathogenicity was monitored using constitutive expression of bacterial luciferase reporter in Salmonella [11]. Fluorescence and bioluminescence imaging each have different advantages and disadvantages depending on the application [12]. Fluorescence enables high-resolution cellular imaging and efficient cell isolation but has limited imaging depths, high background noise, and fluorophores can be photobleached, making quantification more challenging [13]. Bioluminescence is easily quantified due to its minimal background noise and is a direct measure of live cell activity due to its ATP-dependence, making these reporters ideal for non-invasive, real-time tumor monitoring [9, 14]. Consequently, dual bioluminescence and fluorescence reporters have gained interest for their ability to combine the strengths of both techniques.

Early studies using tumor cells expressing bioluminescence reporters were conducted in immunocompromised murine models, which lack a functional host immune system and therefore raised no concerns about immune recognition of the reporter-expressing cells [15, 16]. However, the growing interest in tumor-immune interactions has shifted the focus toward preclinical studies in immunocompetent mouse models, which are essential for understanding the role of the immune system in tumorigenesis and cancer therapeutics [16]. It was reported that GFP has minimal immunogenicity in C57BL/6 immunocompetent mice, however, certain bioluminescence reporters have been found to be immunogenic, potentially complicating their use in such studies [17–19]. Since many reporters are non-mammalian, they are recognized as foreign, non-self antigens [20]. When integrated into syngeneic tumor cells and introduced into an immunocompetent mouse model, these reporters may provoke an immune response against this foreign element [19–21]. For example, dendritic cells could process and cross-present these non-self antigens, leading to the activation of cytotoxic T cells, which then selectively target and eliminate cells expressing the reporter protein [20, 22]. This response could alter the natural course of tumor development, reshape tumor-immune cell interactions, and potentially impact therapeutic efficacy [23]. Identifying a suitable reporter that does not elicit an immune response or cause tumor cell rejection is crucial for studies investigating tumor-immune cell interactions in immunocompetent murine models. Here, we report experimental studies that characterize the immunogenicity of bioluminescence reporters. Our findings demonstrate that the incorporation of click beetle green luciferase does not impact *in vitro* or *in vivo* tumor growth or alter the tumor immune cell composition in preclinical immunocompetent mouse models. This study underscores the importance of characterizing cancer cells stably expressing bioluminescence reporters to ensure that the introduction of the reporter does not alter the tumor-immune microenvironment in syngeneic murine models.

## Results

### Tumor Cells Expressing Red-Shifted Firefly Luciferase Fail to Establish *In Vivo* in Immunocompetent Mice

**Fig. 1:**
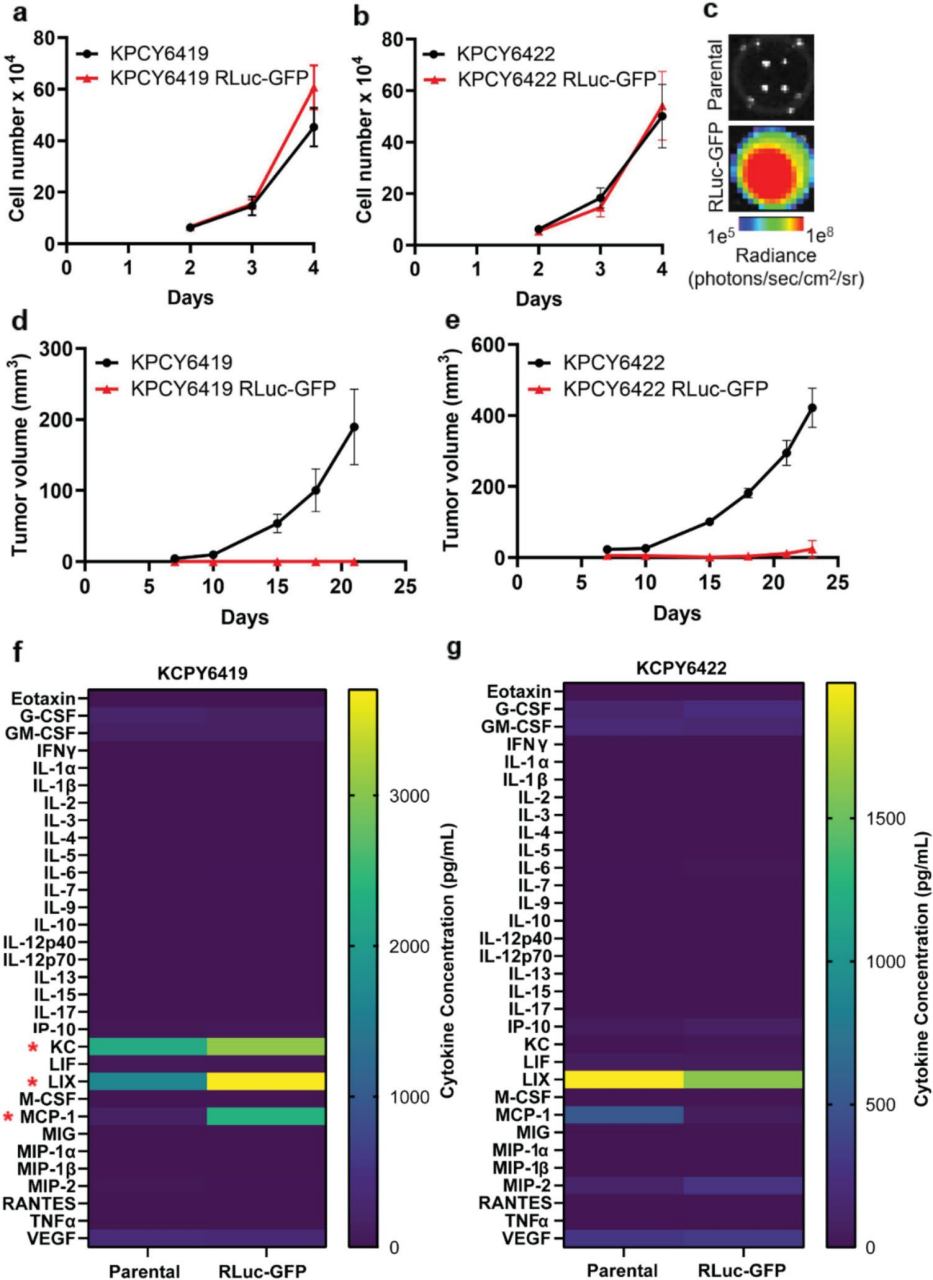
*In vitro* and *in vivo* growth assay of parental and RLuc-GFP PDAC cells. Quantification of cell number over time using a trypan blue viability assay of (**a**) KPCY6419 and (**b**) KPCY6422 parental and RLuc-GFP reporter PDAC cells and (**c**) representative bioluminescence image of KPCY6419 parental and RLuc-GFP cells (*n* = 3 experiments/group). *In vivo* tumor growth curves of (**d**) KPCY6419 and (**e**) KPCY6422 parental and RLuc-GFP reporter PDAC cells (*n* = 3 mice/group). *In vitro* secreted cytokine comparison between (**f**) KPCY6419 and (**g**) KPCY6422 parental and RLuc-GFP reporter PDAC cells (*n* = 3 samples/group). Data shown as mean ± SEM; multiple unpaired t test for cytokine data, * *P* < 0.05

To track tumor cells *in vivo*, the C57BL/6 syngeneic pancreatic ductal adenocarcinoma (PDAC) cell lines, KPCY6419 and KPCY6422, were stably labeled with a red-shifted firefly luciferase and GFP (RLuc-GFP) reporter. No significant differences in *in vitro* growth were observed between the parental and RLuc-GFP PDAC reporter cells (Fig. 1a & b). The *in vitro* expression of RLuc in PDAC cells was confirmed by bioluminescence imaging (Fig. 1c). *In vivo* subcutaneous tumor growth of KPCY parental and RLuc-GFP PDAC cells was evaluated in immunocompetent C57BL/6 mice. Surprisingly, no palpable tumors were observed using either of the PDAC RLuc-GFP cells (Fig. 1d & e). *In vitro* secreted cytokine analysis showed a significant increase in keratinocyte chemoattractant (KC), lipopolysaccharide-induced CXC (LIX) and monocyte chemoattractant protein- 1 (MCP- 1) cytokines in KPCY6419 RLuc-GFP cells compared to KPCY6419 parental cells, while no significant differences in secreted cytokines were observed in KPCY6422 (Fig. 1f & g). The data suggests that while the stable incorporation of the RLuc-GFP reporter did not affect the *in vitro* proliferation of PDAC tumor cells, it did impair the ability of tumor cells to grow *in vivo* in an immunocompetent mouse model.

### Expression of Click Beetle Green Luciferase in Tumor Cells Does not Alter *In Vivo* Tumor Growth

**Fig. 2:**
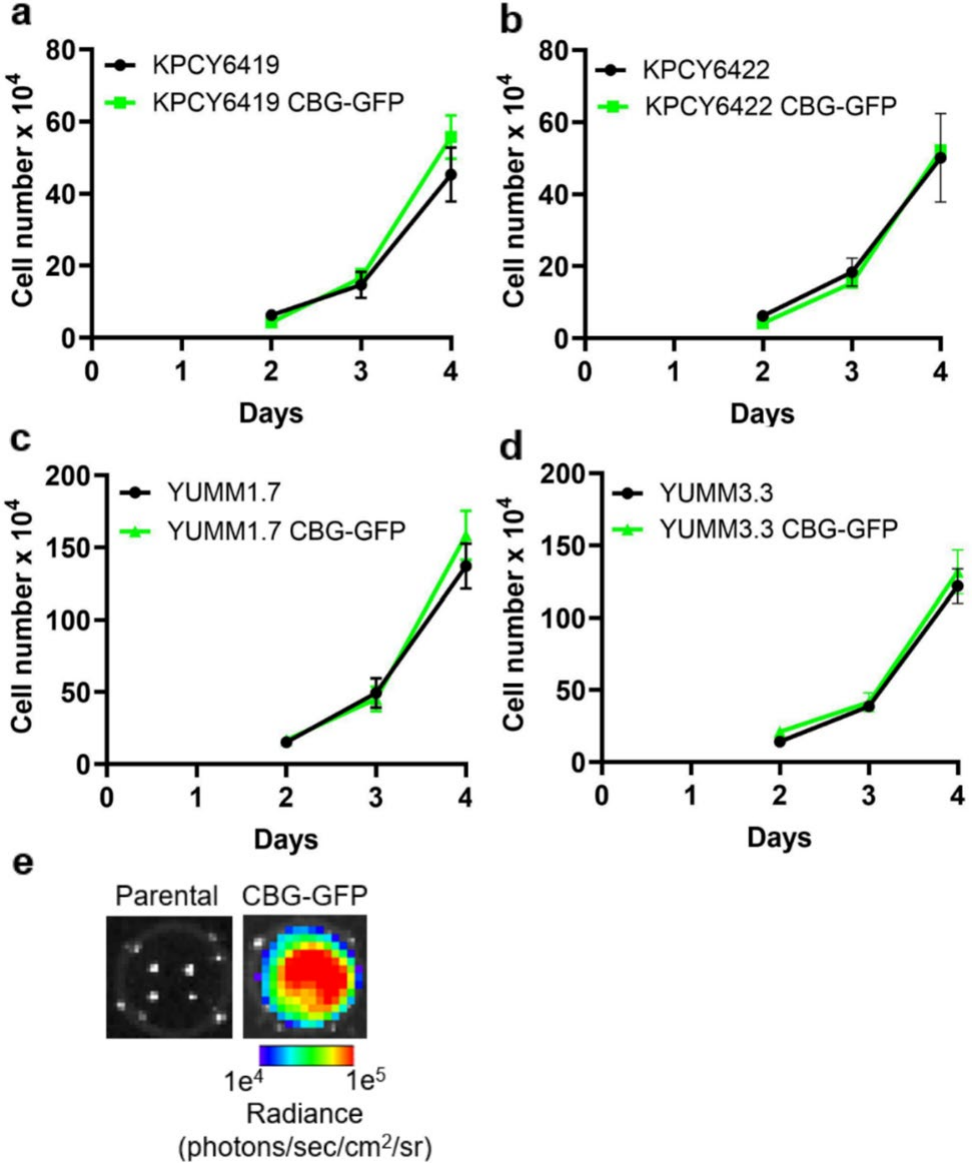
Incorporation of CBG-GFP reporter did not alter *in vitro* cell proliferation of tumor cells. Quantification of the *in vitro* cell number over time comparing parental and CBG-GFP reporter cancer cells: (**a**) KPCY6419, (**b**) KPCY6422, (**c**) YUMM1.7, and (**d**) YUMM3.3 cells. (**e**) Representative bioluminescence image of KPCY6419 parental and CBG-GFP PDAC cells. Data shown as mean ± SEM; unpaired student t test; n = 3 independent experiments/group

Given the lack of *in vivo* tumor growth from RLuc-GFP reporter PDAC cells, we evaluated an alternative bioluminescence reporter, click beetle green luciferase and GFP (CBG-GFP), to track the tumor cells. CBG-GFP was driven by a constitutively active cytomegalovirus (CMV) promoter (Fig. S1). No significant differences in *in vitro* growth were observed between the parental and CBG-GFP reporter PDAC cells (Fig. 2a & b). The incorporation of the CBG-GFP reporter was also evaluated using two melanoma cell lines, YUMM1.7 and YUMM3.3. Similar *in vitro* proliferation rates were observed with the incorporation of the CBG-GFP reporter in melanoma cells (Fig. 2c & d). The expression of luciferase in CBG-GFP reporter cells was confirmed by bioluminescence imaging using D-Luciferin (Fig. 2e). All CBG-GFP reporter cells demonstrated that the CBG bioluminescence signal correlated with cell number (Fig. S2).

**Fig. 3:**
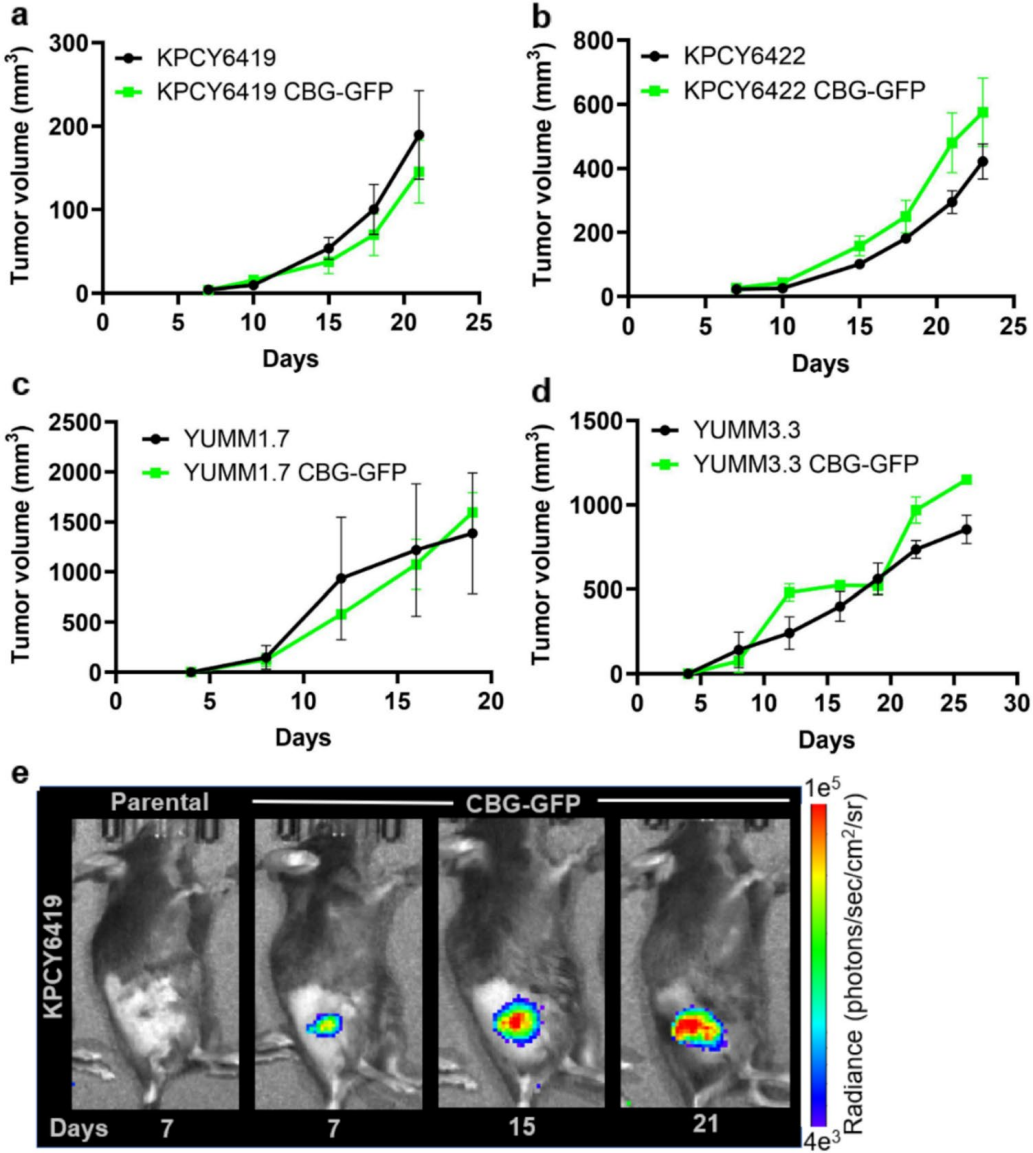
Incorporation of the CBG-GFP reporter did not alter tumor growth. Tumor volume measurements of (**a**) KPCY6419, (**b**) KPCY6422, (**c**) YUMM1.7, and (**d**) YUMM3.3 parental and CBG-GFP reporter cells over time. (**e**) Representative CBG bioluminescence image of KPCY6419 parental and CBG-GFP tumors. Data shown as mean ± SEM; unpaired student t test; *n* = 3–5 mice/group

How the stable expression of the CBG-GFP reporter affected *in vivo* tumor growth was evaluated using subcutaneous tumor models in immunocompetent C57BL/6 mice. Both PDAC and melanoma CBG-GFP cells demonstrated similar *in vivo* growth rates as parental cells (Fig. 3a – d). Tumor CBG-GFP cells were tracked and imaged *in vivo* using CBG bioluminescence throughout cancer growth (Fig. 3e). As expected, tumor CBG bioluminescence increased as the tumor mass increased over time (Fig. S3). We also evaluated a B16F10 melanoma cell line stably expressing the CBG-GFP reporter, driven by a constitutively active ubiquitin promoter [24, 25]. B16F10 CBG-GFP cells exhibited similar *in vitro* proliferation rates and *in vivo* tumor growth compared to parental cells similar to the CMV-driven CBG-GFP cell lines, suggesting that the promoters have minimal immunogenicity (Fig. S4a & b). These data suggest that the CBG-GFP reporter does not interfere with *in vitro* cell proliferation or *in vivo* tumor growth.

### Stable Incorporation of CBG-GFP in Tumor Cell Lines Minimally Alters Secreted Cytokine Profiles

**Fig. 4:**
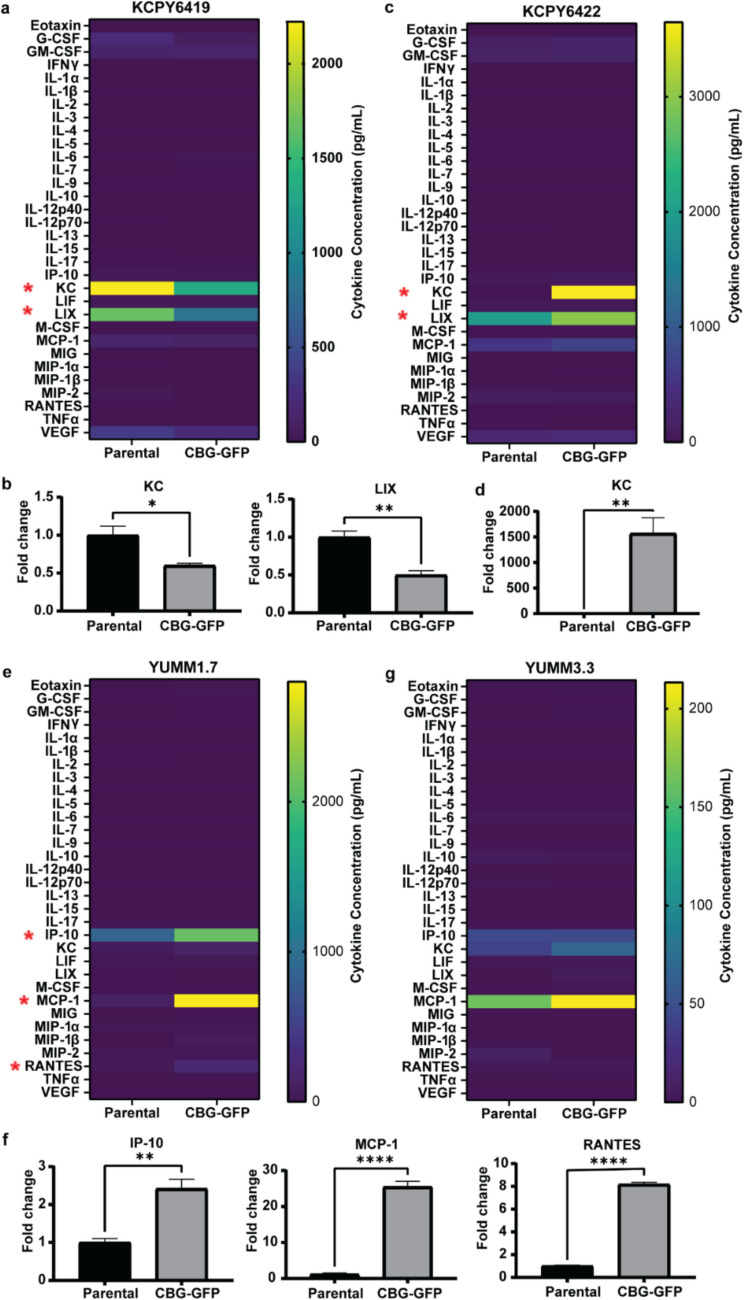
Cytokine expression profiles of parental and CBG-GFP reporter tumor cell lines. Heatmap and corresponding bar graphs of significantly different cytokine levels measured in the supernatant of parental and CBG-GFP (**a**, **b**) KPCY6419, (**c**, **d**) KPCY6422, (**e**, **f**) YUMM1.7, and (**g**) YUMM3.3 cells. Data shown as mean ± SEM; multiple unpaired t-test, * *P* < 0.05, ** *P* < 0.01 **** *P* < 0.0001

How the stable incorporation of CBG-GFP in the cancer cell lines affected the secreted cytokines was evaluated using the supernatants from parental and CBG-GFP reporter cell lines (Fig. 4). In KPCY6419 cells, KC and LIX cytokine levels were reduced (Fig. 4a & b) where CBG-GFP cells had a mean fold change of 0.62 ± 0.11 and 0.50 ± 0.06, respectively. While in KCPY6422 cells, KC levels showed a significant increase with a mean fold change of 2021.32 ± 1482. (Fig. 4c & d). In YUMM1.7 cells, the cytokine analysis revealed a significant increase in levels of interferon-gamma inducible protein- 10 (IP-10), MCP- 1, and regulated upon activation normal T cell expressed and secreted (RANTES) where CBG-GFP cells had a mean fold change of 2.42 ± 0.13, 29.16 ± 10.84 and 8.25 ± 0.59 respectively (Fig. 4e & f). Alternatively, YUMM3.3 cells did not show significant differences in the secreted cytokines between parental and CBG-GFP cells (Fig. 4g). B16F10 CBG-GFP cells demonstrated an increase in IP-10 and RANTES, with mean fold changes of 2.74 ± 0.87 and 2.99 ± 1.22, respectively (Fig. S4 d & e). These data highlight that incorporating an imaging reporter into a tumor cell line can influence cytokine secretion; however, alterations in cytokine secretion do not reliably predict *in vivo* tumor growth.

### No Significant Differences in the Tumor-Immune Composition Between Parental and CBG-GFP Tumors

**Fig. 5:**
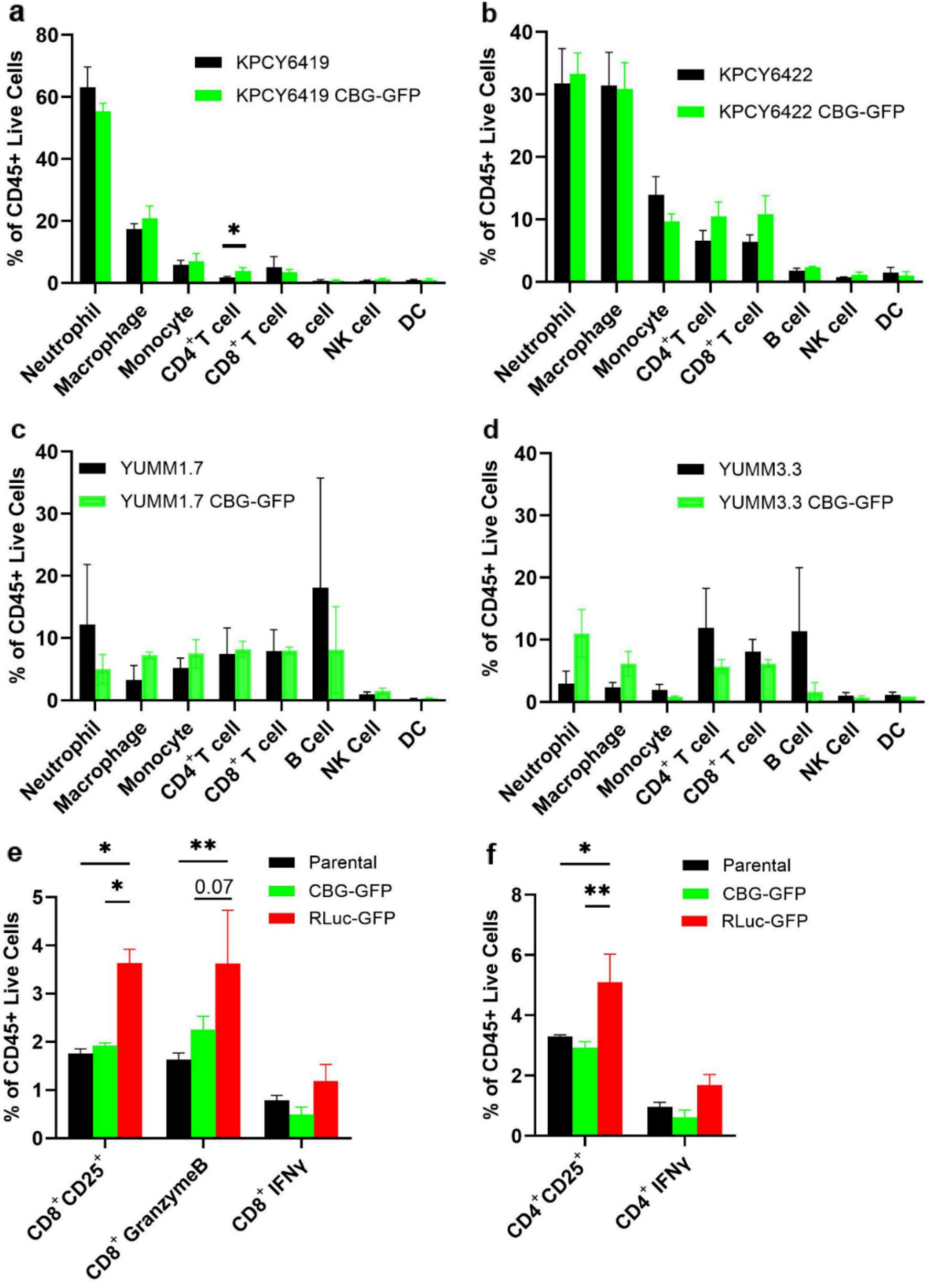
Characterizing the tumor-immune cell composition and activation from reporters. Flow cytometry analysis of immune cell subsets at tumor endpoint in (**a**) KPCY6419, (**b**) KPCY6422, (**c**) YUMM1.7, and (**d**) YUMM3.3 parental and CBG-GFP tumors. Flow cytometry of spleen composition 24 h post inoculation with KPCY6419 parental, KPCY6419 CBG-GFP and KPCY6149 RLuc-GFP cells showing (**e**) CD8^+^ T cell subsets and (**f**) CD4.^+^ T cell subsets. Data are expressed as mean ± SEM; *n* = 3 mice per group, unpaired student t-test or two-way ANOVA followed by Tukey’s multiple comparison test, * *P* < 0.05, ** *P* < 0.01

When studying tumor-immune interactions *in vivo*, it is essential to ensure that the reporter used to track tumor cells does not itself elicit an immune response. Therefore, we evaluated whether the stable incorporation of the CBG-GFP reporter altered the intratumoral immune composition in PDAC and melanoma subcutaneous tumors. No significant differences were observed in the tumor-immune composition of PDAC and melanoma tumors except for KPCY6419 tumors (Fig. 5). A significant 1.76-fold increase in CD4^+^ T cells was observed in KPCY6419 CBG-GFP tumors compared to parental tumors (Fig. 5a). However, the overall percentage of CD4^+^ T cells in KPCY6419 tumors was low, making up approximately 1.7% in parental tumors and 3% in CBG-GFP tumors, respectively. Interestingly, there was a significant increase in monocytes in the B16F10 CBG-GFP tumors compared to the parental tumors, indicating an innate immune response in the B16F10 CBG-GFP tumors that was not evident in the other cell lines (Fig. S4c). Together, these results suggest that generally, the incorporation of CBG-GFP reporter did not alter the tumor-immune microenvironment.

To determine whether an adaptive immune response against RLuc-expressing tumors led to *in vivo* tumor rejection, we assessed T and NK cell activation using flow cytometry and ELISA. Since RLuc-expressing tumors did not grow *in vivo*, we evaluated systemic immune activation using the spleen tissue. A significant increase in activated (CD8^+^ CD25^+^) and cytotoxic (CD8^+^ GranzymeB^+^) CD8^+^ T cells and activated (CD4^+^ CD25^+^) CD4^+^ T cells were observed in mice 24 h post inoculation with KPCY6419 RLuc-GFP cells compared to parental and KPCY6419 CBG-GFP cells (Fig. 5e & f). An increase in CD8^+^ T cells was observed in the spleen of mice injected with KPCY6419 RLuc-GFP cells compared to KPCY6419 CBG-GFP cells (Fig. S5a). A significant decrease in CD4^+^ T cells was observed in the spleen of mice injected with KPCY6419 CBG-GFP cells compared to KPCY6419 RLuc-GFP cells and parental cells (Fig. S5a). No differences were observed in NK cell populations or activation (Fig. S5a). ELISA revealed a trend of increased IFNγ levels but no significant difference in IFNγ or TNFα levels from isolated splenocytes 24 h post-inoculation with KPCY6419 RLuc-GFP cells compared to parental and KPCY6419 CBG-GFP cells (Fig. S5b – e). These data suggest that *in vivo* rejection of RLuc-expressing tumors was due to an increase in CD8^+^ and CD4^+^ T cell activation and cytotoxicity.

### *In Vivo* Applications of Incorporating CBG-GFP Reporter in Cancer Cell Lines

**Fig. 6:**
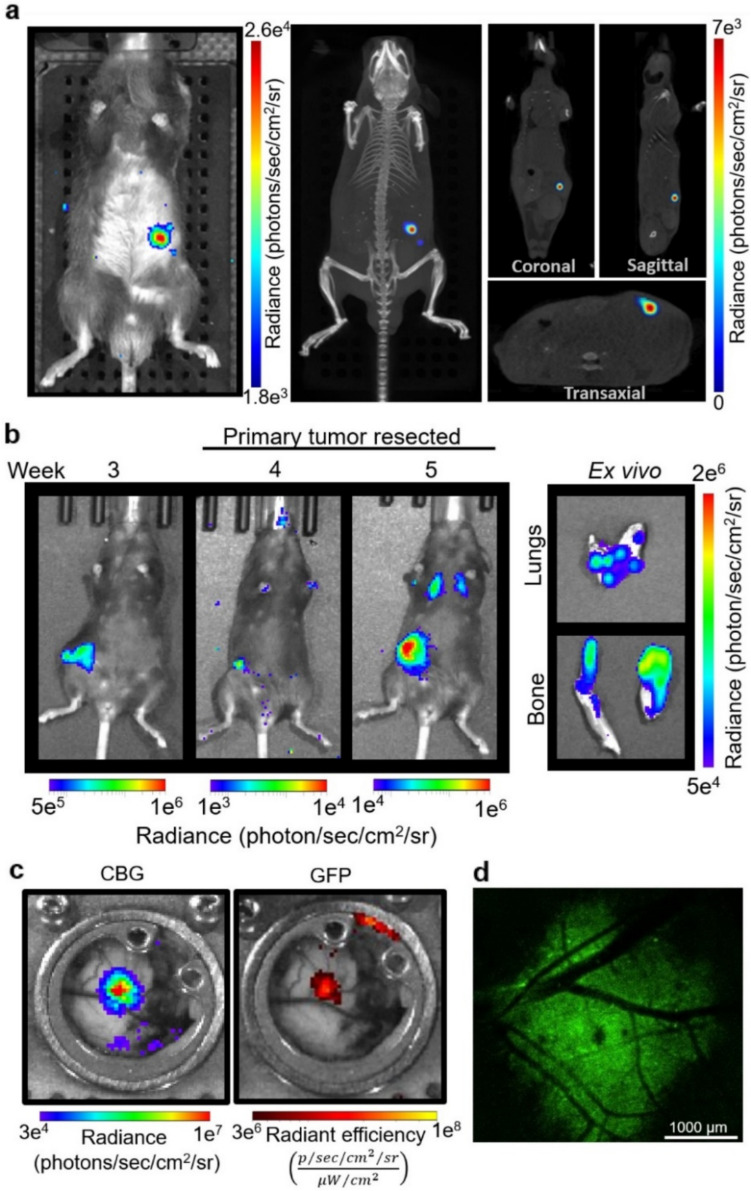
Non-invasive *in vivo* imaging of CBG-GFP cancer cells in various preclinical models. (**a**) Representative 2D bioluminescence (left) and 3D CT (right) images of mice with an orthotopic KPCY6419 CBG-GFP pancreatic tumor on day 15. (**b**) Bioluminescence imaging of the metastatic spread of B16F10 CBG-GFP tumors using a spontaneous metastatic melanoma model. *In vivo* (left) tracking of metastatic spread and e*x vivo* (right) bioluminescence imaging of excised lung and bone metastases. (**c**) Representative macroscopic intravital images of the skin window chamber using YUMM1.7 CBG-GFP tumor-bearing mice. CBG bioluminescence (right) and GFP fluorescence (left) macroscopic imaging of YUMM1.7 CBG-GFP cell. (**d**) Representative intravital confocal image of a skin window chamber using YUMM1.7 CBG-GFP tumor (GFP, green) at 2X magnification

One major advantage of incorporating a CBG-GFP reporter to track cancer cells is the ability to visualize and quantify disease progression non-invasively. The presence and growth of orthotopic pancreatic tumors were monitored following orthotopic implantation of KPCY6419 CBG-GFP cells using 2D bioluminescence and 3D CT imaging (Fig. 6a), showcasing the reporter’s capability for precise localization of tumors within the body. The CBG-GFP reporter was also used to track the metastatic spread of cancer cells. Following subcutaneous tumor resection of B16F10 CBG-GFP melanomas, CBG bioluminescence imaging allowed for non-invasive observation of metastases over time through *in vivo* and *ex vivo* quantification of metastatic burden in the lungs and bones (Fig. 6b). This demonstrates the ability of CBG-GFP to monitor tumor dissemination and metastatic burden in real-time. Macroscopic intravital imaging of YUMM1.7 CBG-GFP tumor-bearing mice within a skin window chamber highlighted both CBG bioluminescence and GFP fluorescence, enabling continuous observation of tumor behavior and interaction with the surrounding microenvironment (Fig. 6c). Detailed visualization of tumor architecture and vascularization was imaged using intravital fluorescence microscopy of the YUMM1.7 CBG-GFP tumor within the skin window chamber (Fig. 6d). These data highlight the versatility of CBG-GFP reporter tumor cells across different cancer models and imaging techniques.

## Discussion

Our studies characterized the immunogenicity of RLuc-GFP and CBG-GFP reporters expressed in cancer cells. Ensuring that the incorporation of imaging reporters do not alter the tumor-immune microenvironment is essential when using immunocompetent murine models, especially for studying tumor-immune interactions. This work presents a strategy to assess whether the stable incorporation of an imaging reporter in tumor cells triggers an immune response. Our data demonstrate that while comparing the *in vitro* proliferation of parental and reporter-expressing cells is useful for confirming stable reporter incorporation, it is insufficient to predict *in vivo* tumor growth. Although no differences in proliferation were observed between parental cells and those expressing RLuc-GFP or CBG-GFP *in vitro* (Fig. 1a & b, Fig. 2), only cell lines expressing CBG-GFP successfully established tumors *in vivo* in immunocompetent mice (Fig. 3). The incorporation of RLuc-GFP in tumor cells resulted in no *in vivo* tumor growth, most likely a result of the immunogenicity of RLuc which elicited an enhanced activation and cytotoxicity of T cells (Fig. 5e & f). Although both PDAC RLuc-GFP cell lines failed to develop tumors *in vivo* (Fig. 1d & e), increased levels of KC, LIX and MCP-1 were observed only in KPCY6419 RLuc-GFP cells compared to parental cells (Fig. 1f). In contrast, no differences in cytokine expression were detected between KPCY6422 parental and RLuc-GFP cells (Fig. 1g). This variability in secreted cytokines suggest that cytokine expression did not contribute to the lack of *in vivo* tumor growth. Similarly, incorporation of the CBG-GFP reporter altered the *in vitro* secreted cytokine expression in several cell lines; a decrease in KC and LIX was observed in KPCY6419 CBG-GFP cells compared to parental cells (Fig. 4b). While an increase in KC was observed in KPCY6422 CBG-GFP cells compared to parental cells (Fig. 4d). An increase in IP-10, MCP-1, and RANTES levels in CBG-GFP cells compared to parental cells (Fig. 4f) while B16F10 CBG-GFP cells had an increase in IP-10 and RANTES (Fig. S4d). KC and LIX are cytokines known to recruit neutrophils and monocytes, with both cytokines binding the CXCR2 receptor [26, 27]. IP-10 and MCP-1 are involved in attracting various immune cells such as T cells, natural killer cells, and dendritic cells to sites of inflammation while RANTES is a cytokine that is involved in the activation and proliferation of T cells [28–30]. However, these changes in secreted cytokines did not correspond to parallel changes in immune cell recruitment to the tumor microenvironment (Fig. 5). Furthermore, no consistent pattern of increased or decreased cytokine levels was observed following the incorporation of the reporter. Similar to RLuc-GFP reporter cells, the incorporation of the CBG-GFP reporter resulted in alterations to a few cytokines; however, CBG-GFP expressing cells demonstrated comparable *in vivo* tumor growth rates and minimal differences in tumor immune cell composition. These findings suggest that the altered cytokine secretion does not significantly impact *in vivo* tumor growth or elicit an immune response. This lack of correlation may stem from the low cytokine concentrations observed *in vitro*, measured in the pg/mL range, or from the host immune system's ability to neutralize and regulate these cytokines *in vivo*, thereby mitigating their effects. These variations in cytokine profiles suggest cell line-specific responses to CBG-GFP expression, yet overall indicate that CBG-GFP does not broadly alter the immune landscape. Thus, *in vitro* proliferation and cytokine analysis alone are insufficient to predict *in vivo* tumor growth and immunogenicity.

How the tumor immune cell composition was affected by the presence of the RLuc-GFP reporter could not be evaluated given the lack of established RLuc-GFP tumors *in vivo*. Several studies have shown that firefly luciferase can provoke an immune response against the reporter protein when used *in vivo* in an immunocompetent mouse [15, 18, 31]. This response may result from the presentation of foreign firefly luciferase antigens to major histocompatibility molecules, which can activate cytotoxic and memory T cell responses. This could enable T cells to eliminate luciferase-expressing tumor cells before they can develop or lead to inconsistent tumor growth patterns [19, 31, 32]. Moreover, a recent study demonstrated that KPC-firefly-luciferase-expressing cells elicited a potent anti-tumor immune response in a PDAC mouse model [18]. This response was characterized by an increase in CD8^+^ T cell and natural killer cell infiltration, resulting in significantly smaller or even absent tumors *in vivo* [18]*.* While previous studies have suggested that GFP may be immunogenic [33, 34], our findings indicate that the immunogenicity observed in our study is primarily attributed to firefly luciferase rather than GFP. This is supported by the fact that CBG-GFP-expressing tumor cells, which also contain GFP, did not provoke a significant immune response or lead to tumor rejection.

Our studies suggest that click beetle green luciferase has minimal immunogenicity, as CBG-GFP-expressing tumor cells exhibited similar *in vivo* tumor growth rates to parental tumor cells. This was consistent across several PDAC and melanoma cell lines in an immunocompetent C57BL/6 mouse model. Minimal differences in tumor immune cell composition were observed with the incorporation of the CBG-GFP reporter in the tumor cells studied (Fig. 5), with the exception of KPCY6419 and B16F10. While a significant increase in intratumoral CD4^+^ T cells was observed in KPCY6419 CBG-GFP tumors compared to parental tumors (Fig. 5a), the overall percentage of CD4^+^ T cells remained low, and no other significant changes in immune composition were noted. A significant increase in monocytes was observed in B16 F10 CBG-GFP tumors compared to parental tumors (Fig. S4e). However, no other significant changes in immune cells were observed. Given the absence of differences in *in vivo* tumor growth, the single alteration in immune cell subsets resulting from the incorporation of CBG-GFP into KPCY6419 and B16F10 cells is unlikely to significantly impact future studies evaluating tumor-immune interactions during tumor development. Collectively, these findings highlight the minimal immunogenic effects of CBG-GFP expression in tumor cells demonstrating its suitability as an optimal reporter for preclinical tumor immunology studies.

While this study provides valuable insights into the use of bioluminescence reporters, several limitations must be acknowledged. One significant limitation is that the immune composition of tumors was only evaluated at tumor endpoint. Evaluating immune composition only at the endpoint may miss early immune responses critical for understanding tumor-host interactions and influencing tumor progression [35]. Although no differences in tumor growth were observed, the potential impact of CBG-GFP incorporation on early immune events cannot be excluded. Future studies should incorporate temporal analysis of immune responses following tumor inoculation to more comprehensively understand immune dynamics. Additionally, loss of reporter plasmids represents another limitation, as observed in Pan02 PDAC cells labeled with the CBG-GFP reporter (Fig. S6). This issue resulted in the formation of palpable tumors with similar *in vivo* tumor growth rates but lacked a bioluminescence signal, even after three *in vivo* selection passages. This highlights the cell line-specific variability in response to CBG-GFP expression and emphasizes the need for developing more stable integration methods to ensure consistent expression throughout *in vivo* studies. While CBG-GFP showed minimal immunogenicity in C57BL/6 mice, its immunogenic profile in other mouse models remains uncertain. Variations in tumor microenvironments and host genetic backgrounds may significantly shape the immune response to reporter genes. While our data demonstrate the applicability of CBG-GFP in five cancer cell lines, we were unable to establish a stable Pan02 CBG-GFP cell line. Additionally, lentiviral infection results in the random integration of reporter genes, which can disrupt cellular pathways and alter cell behavior, potentially increasing immunogenicity depending on the cell line and mouse model. These studies utilized polyclonal reporter cell populations; isolating monoclones may reveal differences in immunogenicity. Future studies will assess the immunogenicity of these monoclones. Therefore, it is essential to validate the stability and lack of immunogenicity of the CBG-GFP reporter when incorporating it into new cancer models. Lastly, our study did not evaluate the immunogenicity of GFP. However, GFP has been widely used in cancer models since the 1990 s and is well-established for tracking tumor cells *in vivo* [36, 37]. Our focus was on the impact of bioluminescence reporters within dual bioluminescence-fluorescence systems. These dual reporters offer several advantages; GFP facilitates the efficient isolation of labeled tumor cells, a process that is challenging when relying solely on bioluminescence reporters and eliminates the need for antibiotic selection genes. Furthermore, dual reporters synergistically combine the strengths of both imaging modalities. GFP allows for high-resolution cellular microscopy, though its effectiveness is constrained by limited tissue penetration and potential phototoxicity. In contrast, bioluminescence imaging provides quantitative capabilities, enhanced sensitivity, minimal background noise, and the ability to perform real-time, non-invasive, longitudinal whole-body imaging. Together, these features make dual reporters highly versatile tools for studying tumor immunology.

## Conclusion

This study highlights the importance of evaluating the immunogenicity of imaging reports for *in vivo* tumor studies. The use of dual bioluminescence and fluorescence reporters, such as CBG-GFP, offers significant advantages by enabling complementary imaging techniques that combine the deep tissue imaging capability of bioluminescence with the high-resolution tracking of fluorescence. While the incorporation of the RLuc-GFP reporter in cancer cells resulted in a lack of tumor growth *in vivo*, CBG-GFP demonstrated minimal immunogenicity with comparable tumor growth to parental tumor cells. Therefore, careful selection of the bioluminescence reporter used to track tumor progression is essential. Specific experiments should be conducted to ensure that the incorporation of the imaging reporter does not interfere with tumor growth or alter the tumor-immune composition of the cancer model. This work emphasizes the critical need for initial assessment of *in vivo* effects of reporters to ensure the successful and effective development of reporter tumor cells to study tumor biology, immunology and assess cancer therapeutics.

## Supplementary Information

Below is the link to the electronic supplementary material.

Supplementary file1 (DOCX 2.09 MB)

## Data Availability

All data relevant to the study are included in the article or uploaded as supplementary materials.

## Declarations

### Materials and Methods

Available in supplementary materials.
