# Supplementary material for: Immune Response to Bioluminescence Imaging Reporters in Murine Tumor Models
Source: Mol Imaging Biol. 2025 Apr 15;27(3):341–52. doi: 10.1007/s11307-025-02010-7 (PMC12162783; doi:10.1007/s11307-025-02010-7)
Supplement: Supplementary file 1 — Supplementary file1 (DOCX 2.09 MB) [file 11307_2025_2010_MOESM1_ESM.docx]

**Supplementary Materials**

**Immune response to bioluminescence imaging reporters in murine tumor models.**

Angisha Basnet^1^, Dylan D. Thomas^1^, Kaitlyn M. Landreth^1^, F. Heath Damron^1,2^, Tracy W. Liu^1, 3^ *

**Affiliations:**

^1^ Department of Microbiology, Immunology, and Cell Biology, West Virginia University, Morgantown, WV, 26506, USA.

^2^ Vaccine Development Center, West Virginia University Health Sciences Center, Morgantown, WV, 26505, USA.

^3^ WVU Cancer Institute, West Virginia University, Morgantown, WV, 26506, USA.

* Corresponding author:

Tracy W. Liu, Ph.D.,

Department of Microbiology, Immunology, and Cell Biology

School of Medicine

West Virginia University

64 Medical Center Drive

Morgantown, WV, 26506

Tel: 304-293-3104

Fax: 304-293-7823

Email: [tracy.liu@hsc.wvu.edu](mailto:tracy.liu@hsc.wvu.edu)

**Materials and Methods**

Cell lines: KPCY6419 and KPCY6422 (Kerafast Inc., Boston, MA) were cultured in Dulbecco’s modified Eagle’s medium (DMEM) supplemented with 10% heat-inactivated fetal bovine serum (FBS). YUMM1.7 and YUMM3.3 cell lines (ATCC, Manassas, VA) were cultured in DMEM F12 medium supplemented with 10% heat inactivated FBS and 1% non-essential amino acids (NEAA). B16F10 cells (ATCC, Manassas, VA) were cultured in DMEM supplemented with 10% heat-inactivated FBS. The Pan02 cell line (Division of Cancer Treatment and Diagnosis Tumor Repository at the National Cancer Institute, Bethesda, MD, USA) was grown in RPMI-1640 media with 10% heat inactivated FBS. All cell cultures were grown at 37°C in 5% CO2. 0.25% Trypsin-EDTA (1X) was used to detach the cells from culture flasks. All cell lines tested negative for mycoplasma.

Reagents: D-Luciferin, Potassium Salt was purchased from Gold Biotechnology Inc. (St. Louis, MO, USA). D-Luciferin was dissolved in sterile phosphate-buffered saline (PBS) to a final concentration of 15 mg/ml, passed through a syringe filter of 0.45 µm, and stored at −20°C. All fluorophore conjugated antibodies (Table S1) were purchased from BioLegend (CA, USA) except CD11b-APC, which was purchased from Miltenyi Biotec, Inc. (CA, USA). Polybrene was purchased from Vector builder Inc. (Chicago, IL, USA). Penicillin-streptomycin solution was purchased from Cytiva (Utah, USA). Fetal Bovine Serum (FBS) was purchased from VWR International, LLC. (PA, USA). Trypan blue (0.4%), Penicillin-Streptomycin (10,000 U/mL), NEAA and 0.25% Trypsin-EDTA (1X) were purchased from Life technologies corporation (Gibco, NY, USA). Sodium pyruvate was purchased from Mediatech, Inc. (Manassas, VA, USA).

Reporter plasmids: The red-shifted *Luciola Italica* luciferase and green fluorescent protein (RLuc-GFP) lentiviral particle was purchased from Perkin Elmer (Waltham, MA). The click beetle green luciferase and green fluorescent protein (CBG-GFP) plasmid was built and packaged into a lentiviral particle by Vector Builder (Chicago, IL, USA). The plasmid map is shown in Figure S1.

Generation of reporter cells: To generate cell lines stably expressing CBG-GFP or RLuc-GFP, 2 x 10^5^ cells were plated in a 6-well plate and incubated overnight at 37°C in a humidified 5% CO₂ atmosphere. Once cells reached 30–50% confluence, they were infected with CBG-GFP lentivirus or RLuc-GFP lentiviral particle at an MOI of 10 in the presence of 5 µg/mL polybrene for CBG-GFP and 4 µg/mL polybrene for RLuc-GFP. After 48 hours, cells were trypsinized, expanded in T-75 flasks, and at approximately 80-90% confluency selected for GFP expression via fluorescence-activated cell sorting (FACS). The top 20% of GFP-expressing cells were expanded and then sorted a second time by FACS to isolate the top 20%. For bioluminescence imaging, isolated cells were cultured in phenol-free DMEM containing 10% FBS and 150 µg/mL D-Luciferin, and bioluminescence was confirmed using the Kino imaging system (Spectral Instruments Imaging, AZ, USA) for CBG-GFP cells and IVIS spectrum (Perkin Elmer, Waltham, MA) for RLuc-GFP cells using the following acquisition parameters: acquisition time, auto; binning, 8; FOV, 13.2; f/stop, 1; filter, open.

*In vitro* growth assay: 1 x 10^4^ parental and reporter cells (Rluc-GFP and CBG-GFP) were seeded in 6-well plates. On day 2, 3 and 4 post seeding, cells were trypsinized and cell numbers were counted using trypan blue on a cellometer (Nexcelom Bioscience LLC, Lawrence, MA, USA).

*In vivo* subcutaneous tumor model: All animal protocols were approved by the Institutional Animal Care and Use Committee at West Virginia University (protocol #2109047227). Female C57BL/6 mice (8 weeks old, The Jackson Laboratory, Bar Harbor, ME) were injected subcutaneously on the right flank with 2 x 10^5^ KPCY6419 and KPCY6422 cells (parental, RLuc-GFP, or CBG-GFP), 1 x 10^5^ YUMM1.7 and YUMM3.3 cells, or 5 x 10^4^ B16F10 cells (parental or CBG-GFP). Tumor growth was monitored twice weekly using calipers once tumors became palpable. *In vivo* bioluminescence imaging was performed 9 minutes after intraperitoneal injection of D-luciferin (150 mg/kg) using an IVIS spectrum with standard acquisition settings. Mice were euthanized following institutional guidelines when tumors reached 1.5 cm in diameter or ulcerated beyond 0.5 cm.
*In vivo* Pan02 CBG-GFP cell sorting: 5 x 10^5^ Pan02 CBG-GFP cells were injected on the right flank of 8-week-old female C57BL/6 mice. D-luciferin bioluminescence image was taken weekly as previously described. Not all tumors maintained expression of the CBG-GFP reporters. Thus, at tumor endpoint, tumors with the highest bioluminescence were excised, made into a single cell suspension, and cultured with RPMI-1640 media (10% FBS and 1X penicillin-streptomycin). At 90% confluency, these *in vivo* selected Pan02 CBG-GFP cells were FACs sorted where the highest 20% were isolated and expanded in a T-175 flask. These sorted cells were injected into the right flank of mice and the *in vivo* selection process was repeated three cycles.

Flow cytometry: At tumor endpoint, animals were euthanized by carbon dioxide asphyxiation. Single-cell suspension of cells was harvested from the tumor. Tumors were dissociated using a mouse tumor dissociation kit (Miltenyi Biotec, CA, USA). Cells were passed through a 70 µm sterile cell strainer, centrifuge at 300 rpm for 7 minutes and resuspended in cell staining buffer (BioLegend, CA, USA) at a concentration of 1 x 10^6^ cells per 100 µl. Spleen was harvested from KPCY6419 parental, CBG-GFP and RLuc-GFP mice 24-hour post tumor injection. A single cell suspension was made by gently smashing spleen using a plunger in a cell strainer with buffer. Red blood cell (RBC) lysis was performed by incubating the cells with 3 mL of 1x lysis buffer (Thermo Fisher Scientific, CA, USA) on ice for 5 minutes. After RBC lysis, the remaining cells were counted and resuspended in cell staining buffer (BioLegend, CA, USA) at a concentration of 1 × 10^6^ cells per 100 µl. Fc receptors were blocked using 10 µg/ml ChromPure of mouse IgG antibody (Jackson ImmunoResearch Inc., PA, USA) per 10^6^cells in a 100 µl volume for 10 minutes on ice. Cells were washed with cell staining buffer and incubated with the antibody mix (Table S1 for tumor cell staining and table S2 for spleen cells staining) for 15 – 20 minutes on ice in the dark. Cells were fixed using 200 µl fixation buffer (Thermo Fisher Scientific, CA, USA) for 20 min at 4ºC in the dark. Cells were washed 2 times with cell staining buffer and were analyzed using the Cytek® Aurora (Cytek, MD, USA) within 2 weeks of staining. Data was analyzed using FCS express (De Novo Software, CA, USA).

ELISA: Splenocytes were harvested from KPCY6419 parental, CBG-GFP, and RLuc-GFP mice 24 hours post-tumor injection. After red blood cell (RBC) lysis, the cells were cultured for 24 hours at a concentration of 4 x 10⁵ cells per 200 µL in a 96-well plate. The culture supernatant was then collected and stored for subsequent ELISA analysis. Blood samples were collected intracardially from the same groups of mice and centrifuged at 1,000 × g for 10 minutes. The serum was collected and used for ELISA. The manufacturer's kit protocol was followed to quantify IFN-γ (mouse IFN-γ ELISA kit, Invitrogen, CA, USA) and TNF-α (mouse TNF-α ELISA kit, Invitrogen, CA, USA) levels in both the serum and culture supernatants.

Cytokine analysis: 7.5 x 10^4^ cells were seeded in 500 μl of media in 24 well plates for 48 hours. After 48 hours, supernatant was collected and centrifuged at 3000 g at 4° C for 5 minutes to remove cellular debris and stored at -80°C. Cytokine quantification was performed using the Luminex xMAP technology on a Luminex™ 200 system (Luminex, Austin, TX, USA), facilitated by Eve Technologies Corp (Calgary, AB, Canada). A total of 32 mouse cytokines, chemokines, and growth factors were simultaneously measured using the Mouse Cytokine 32-Plex Discovery Assay® from MilliporeSigma (Burlington, Massachusetts, USA). The assay sensitivities ranged from 0.3 to 30.6 pg/mL. Fold change was calculated by normalizing each cytokine data with the average cytokine data of the parental cells.

Orthotopic pancreatic cancer model: Mice were anesthetized using isoflurane and the surgical area was prepared using aseptic techniques. A small incision was made on the left abdomen to expose the pancreas. 10 µl of 2 x 10^5^ of KPCY6419 CBG-GFP cells mixed with matrigel in 1:1 ratio was injected directly into tail of pancreas using an insulin syringe. After tumor cell implantation, the pancreas and spleen were carefully placed back into the peritoneal cavity, and the abdominal muscle layer and the skin layer were sequentially sutured closed. At day 15 post tumor implantation, 3D bioluminescence CT imaging was done 9 min post D-luciferin (150 mg/kg body weight) administration using an IVIS spectrum using the following acquisition parameters: acquisition time, 1 min; binning, 8; FOV, 13.2 f/stop, 1; filter, open.

Melanoma metastasis model: 5 x 10^4^ B16F10 reporter cells were prepared at a 1:1 ratio with Matrigel to a total volume of 100 µL and subcutaneously injected into the lower back of 8-week old C57BL/6 mice. Tumor growth was monitored bi-weekly until tumors reached a target size of 8-10 mm, where tumors were then surgically resected under sterile conditions and anesthesia using isoflurane. Weekly bioluminescence imaging, 9 min post D-luciferin (150 mg/kg body weight) administration using an IVIS spectrum, post subcutaneous tumor resection monitored tumor metastatic spread. At week 6, mice were euthanized and lungs and femurs were removed for *ex vivo* bioluminescence imaging.

Skin window chamber: Skin window chamber implantation and imaging were previously described [24-25]. Following skin window chamber implantation, inoculation of 1 x 10^6^ YUMM1.7 CBG-GFP cells in 10 μL was implanted in C57BL/6 mice. Nikon confocal AX microscopy (Nikon, NY, USA) occurred at day 3 post skin window chamber implantation. Tumors were visualized within the skin window chamber using GFP confocal imaging at 2X magnification. Macro-imaging occurred at day 6 post implantation using the IVIS spectrum (Perkin Elmer, Waltham, MA) as described above in the *in vivo* subcutaneous tumor model section. Macro fluorescence imaging of tumors stably expressing GFP was performed prior to bioluminescence imaging.

Statistical analyses: Graphs were made and statistical analyses were performed using GraphPad Prism (GraphPad Software, Inc, CA, USA). Data were expressed as mean ± SEM. Analysis of differences between two normally distributed paired test groups was performed using a student’s t-test. For analysis of three or more groups, analysis of variance (ANOVA) tests was performed followed by a multiple comparisons test. P values were considered statistically significant if *P* < 0.05.


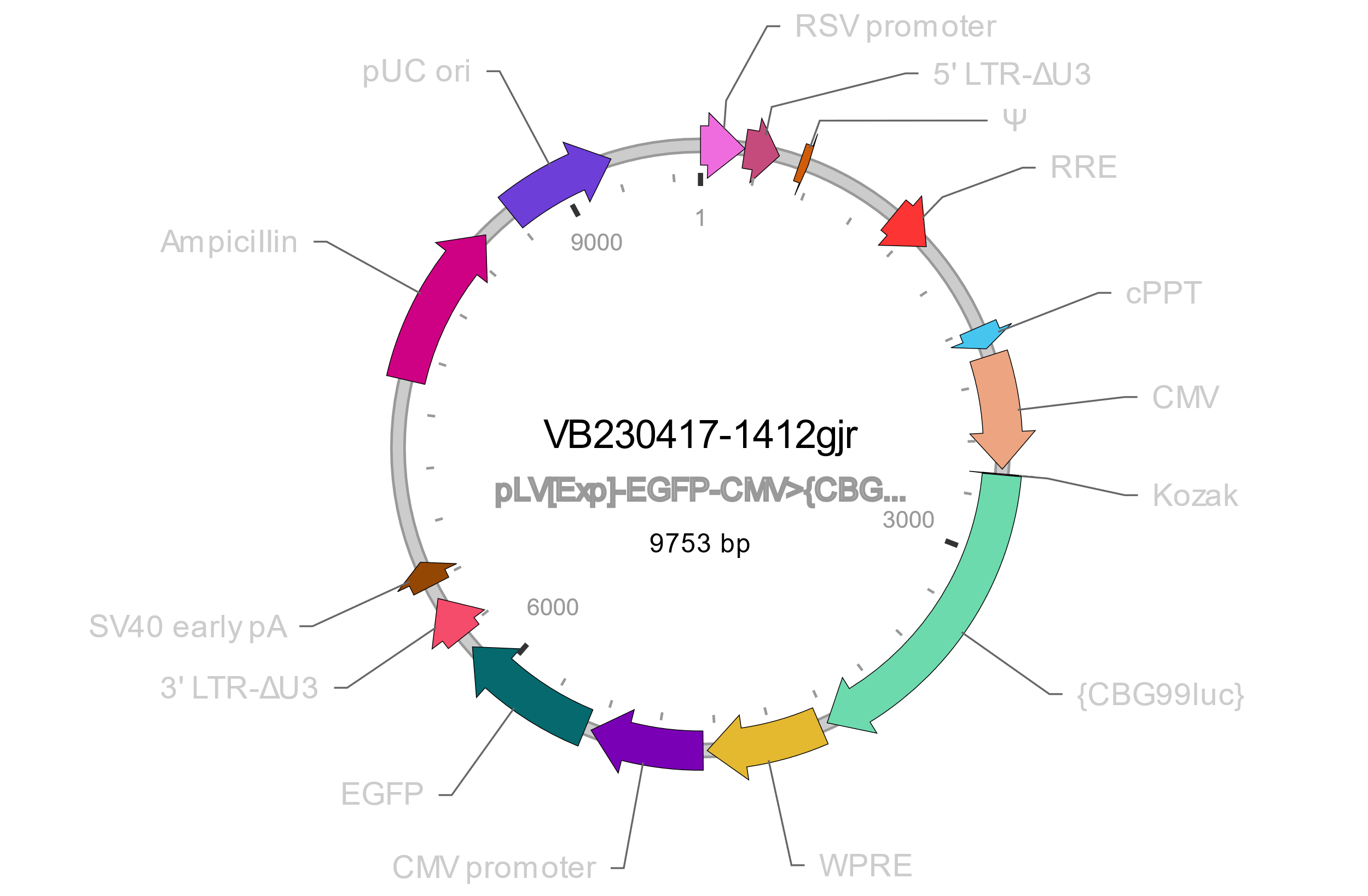
Figure S1. Plasmid map for click beetle green luciferase and green fluorescent protein (CBG-GFP) reporter.


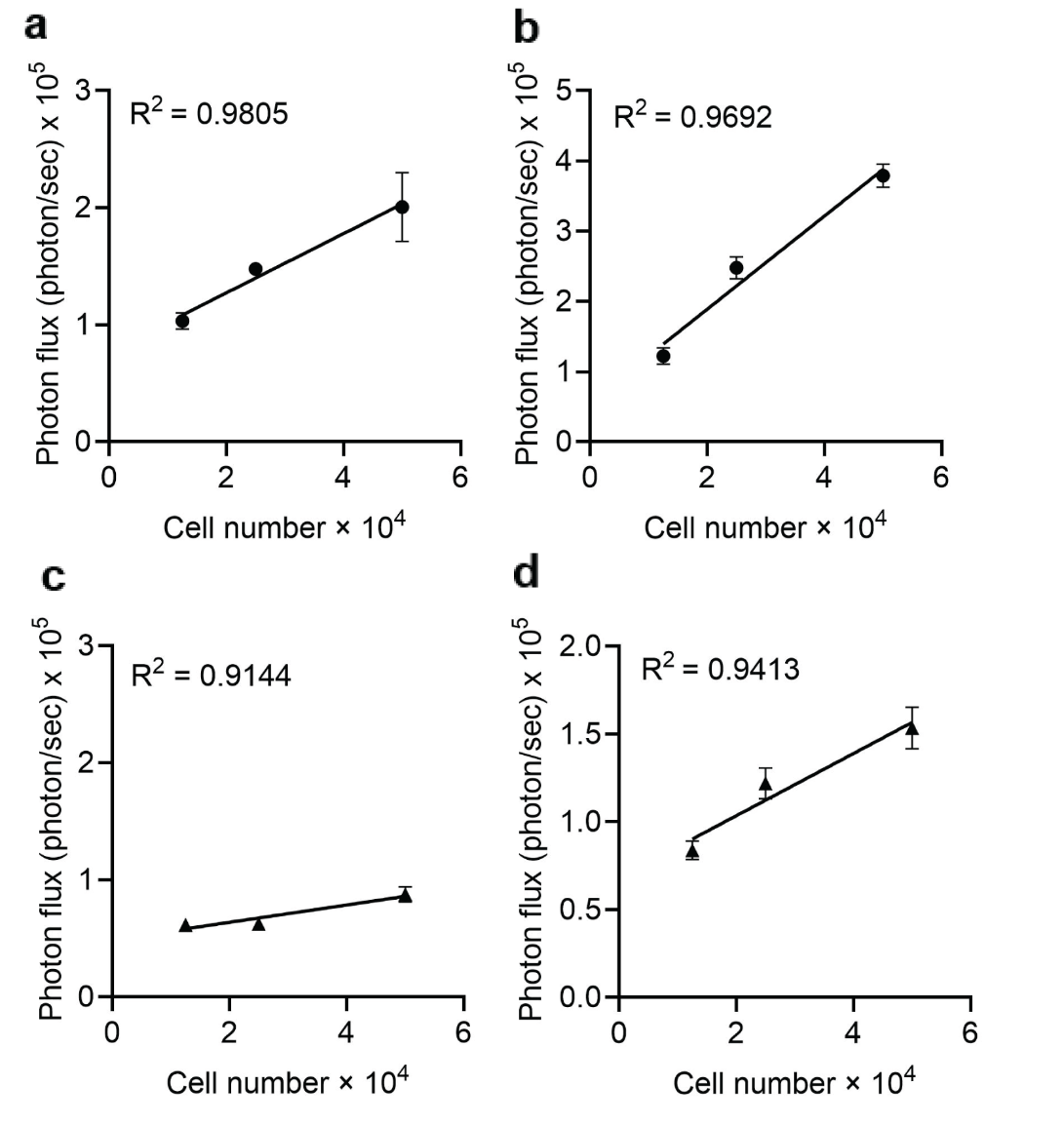


Figure S2. Correlation of (a) KPCY6419, (b) KPCY6422, (c) YUMM1.7, and (d) YUMM3.3 CBG-GFP bioluminescence signal with cell number. n= 3 individual experiment per group. Data are shown as mean ± SEM, and R-squared value were determined by simple linear regression analysis.


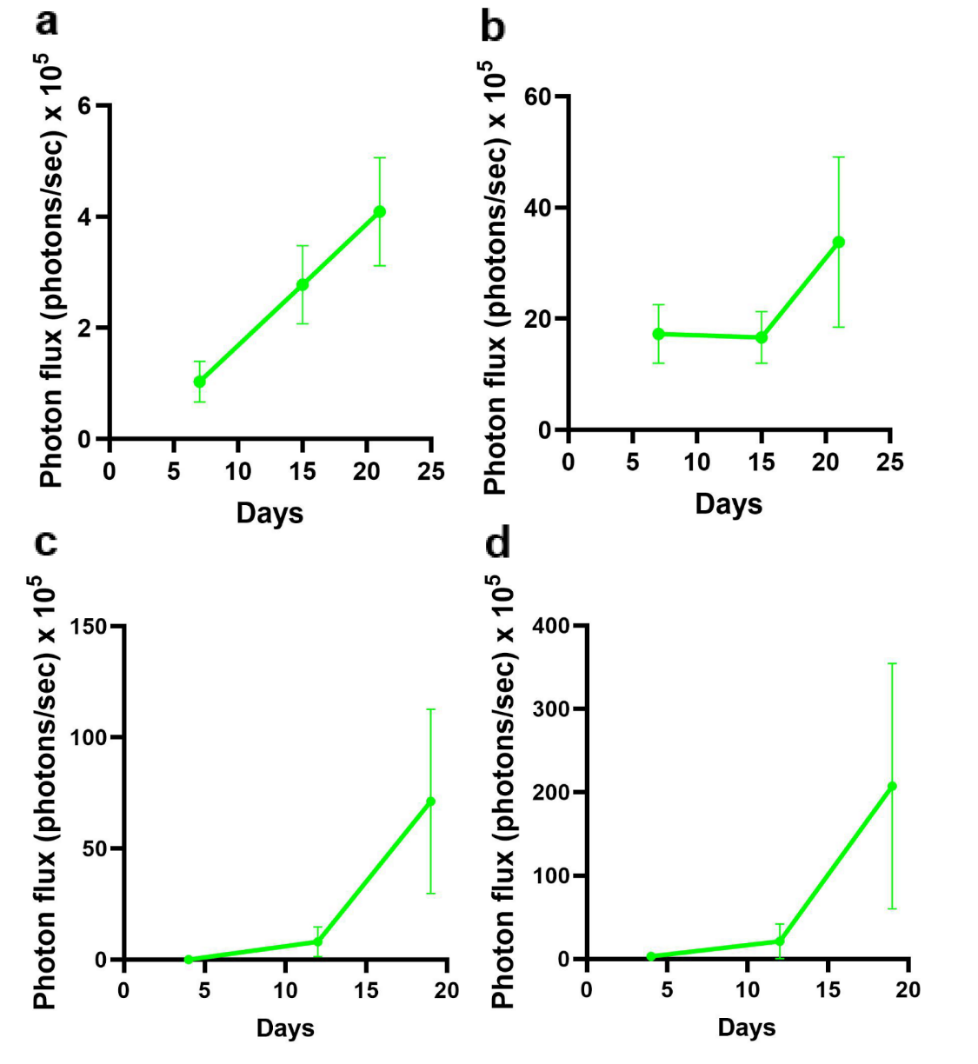


Figure S3. Quantification of *in vivo* tumor CBG bioluminescence of (a) KPCY6419 CBG-GFP, (b) KPCY6422 CBG-GFP, (c) YUMM1.7 CBG-GFP, and (d) YUMM3.3 CBG-GFP. Data shown as mean ± SEM, n= 3 – 5 animal/group.


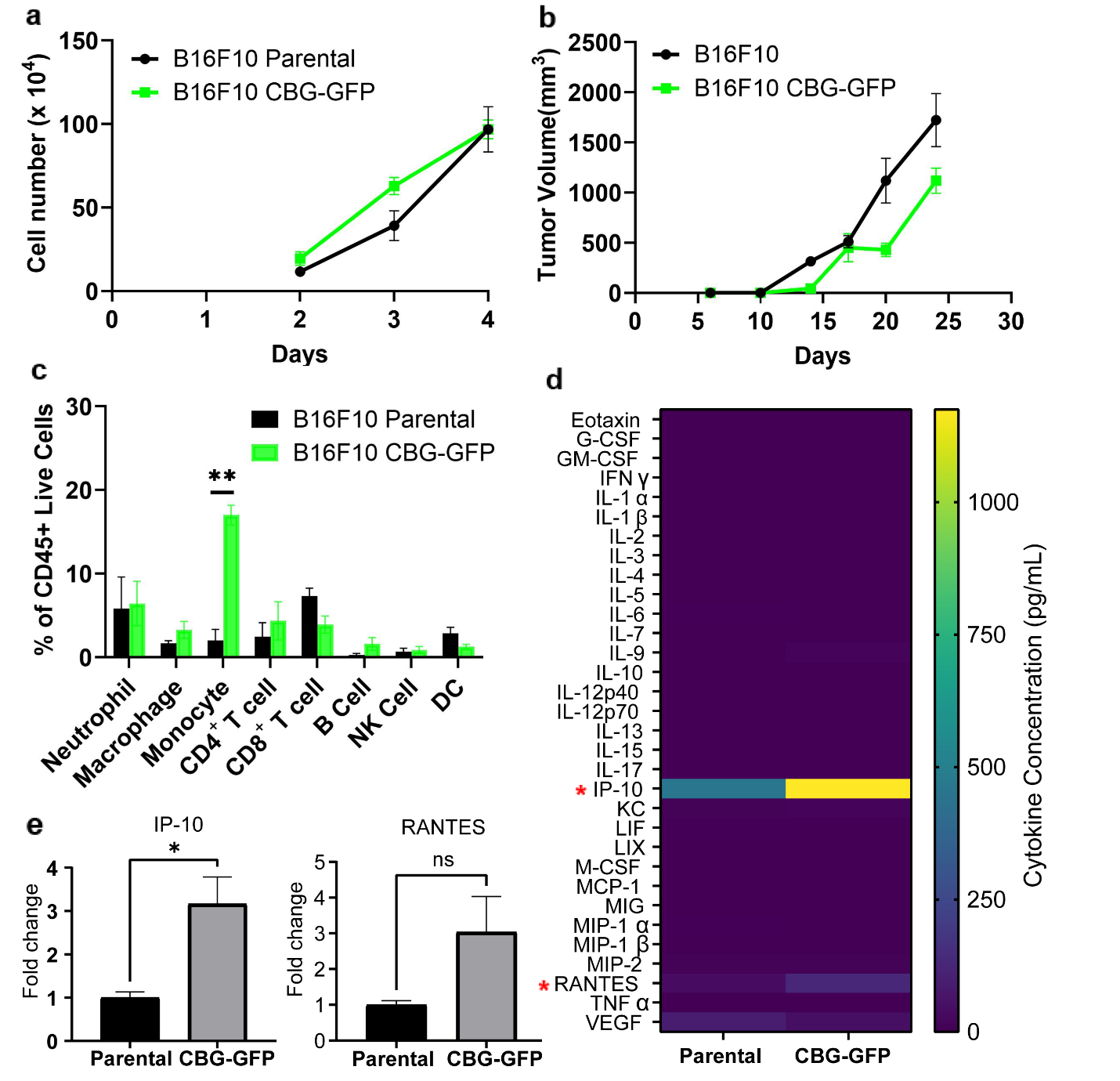


Figure S4: Comparative analysis of B16F10 parental and CBG-GFP reporter cells. (a) *In vitro* cell proliferation of B16F10 parental and CBG-GFP reporter cells. (b) *In vivo* tumor growth curves of B16F10 parental and CBG-GFP reporter cells. (c) Tumor immune cell composition at endpoint of B16F10 parental and CBG-GFP tumors characterized by flow cytometry (d, e) Heatmap and corresponding bar graphs of significantly different cytokine levels measured in the supernatant of B16F10 parental and CBG-GFP reporter cells. Data presented as mean ± SEM; n=3 mice/group; multiple t-tests; * *P* <0.05, ** P<0.01.


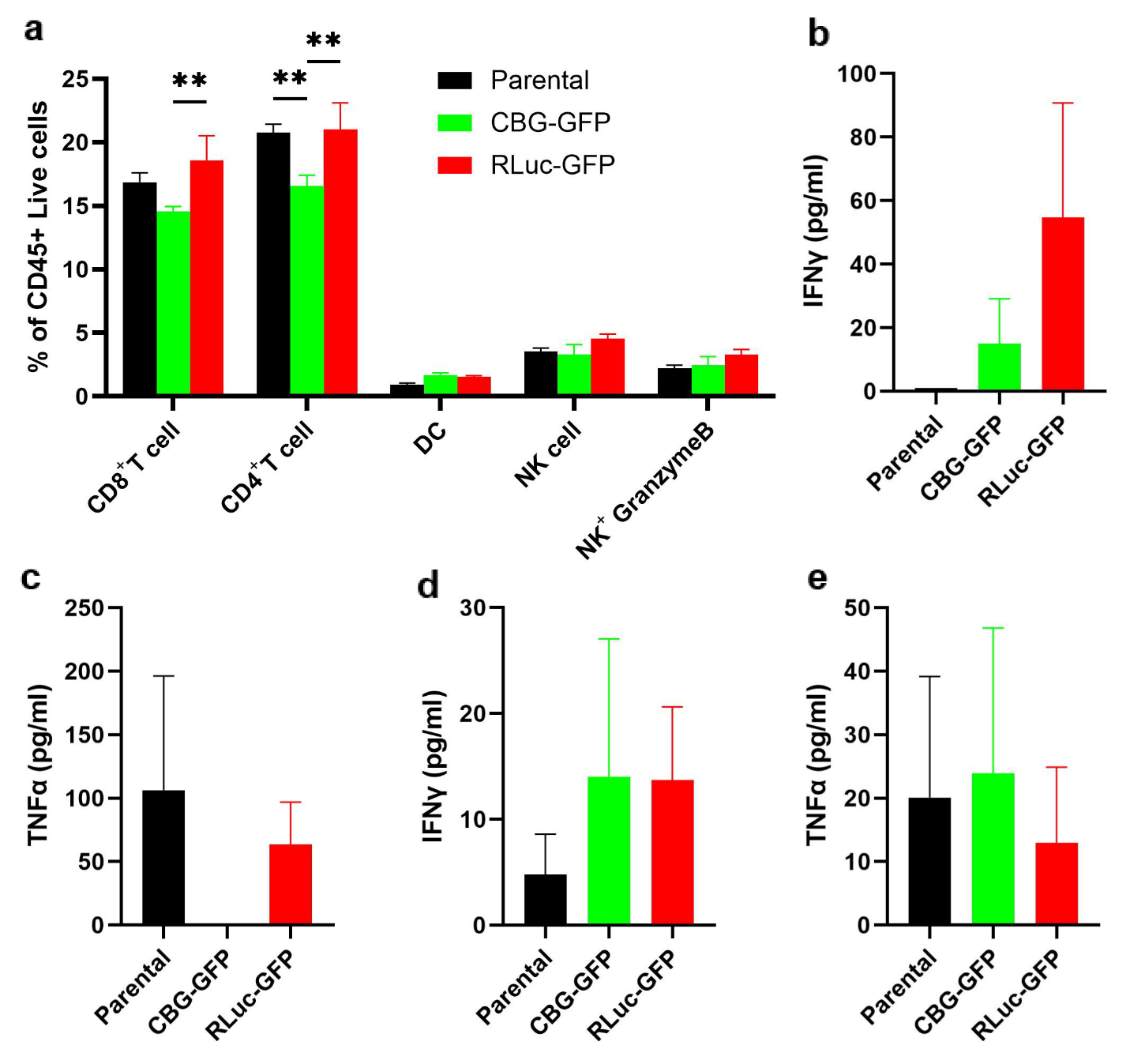


Figure S5. Comparative analysis of spleen-immune cell composition and cytokine levels from mice injected with KPCY6419 parental, CBG-GFP or RLuc-GFP. (a) Flow cytometry of the immune cells composition of the spleen at 24 hours post inoculation with KPCY6419 parental, CBG-GFP and RLuc-GFP cells. ELISA data showing (b) IFNγ and (c) TNFα levels in the supernatant of isolated splenocytes after 24 hours in culture and (d) IFNγ and (e) TNFα levels from the serum of KPCY6419 parental, CBG-GFP and RLuc-GFP mice 24 hours post inoculation. Data are expressed as mean ± SEM; n = 3 mice per group, two-way ANOVA for spleen immune cell composition and one-way ANOVA for ELISA data followed by Tukey’s multiple comparison test, ** P<0.01.


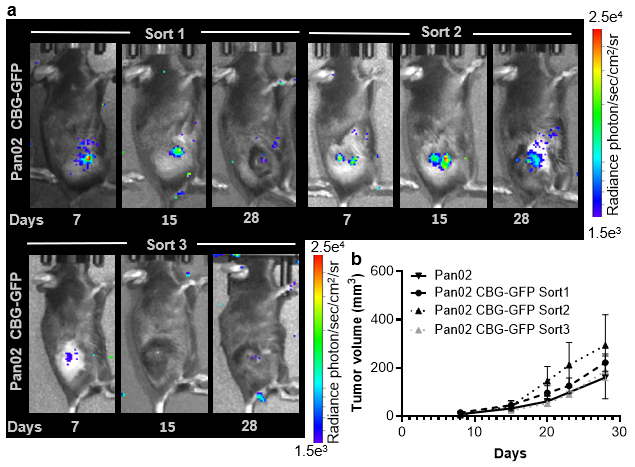


Figure S6. (a) Representative CBG bioluminescence image of Pan02 CBG-GFP tumors from multiple *in vivo* sorted Pan02 CBG-GFP cells. (b) Tumor volume comparison of multiple Pan02 parental and CBG-GFP sorted cells. Data shown as mean ± SEM; unpaired student t test; n = 3 – 5 mice/group.

| **Antibody** | **Company** |
| --- | --- |
| CD45-Pacific blue | BioLegend (CA, USA) |
| CD3-Alexa Fluor 488 | BioLegend (CA, USA) |
| CD4-BV 785 | BioLegend (CA, USA) |
| CD8-APC Fire 750 | BioLegend (CA, USA) |
| CD19-APC Fire 810 | BioLegend (CA, USA) |
| CD11c-BV 421 | BioLegend (CA, USA) |
| CD45R-Spark Blue 550 | BioLegend (CA, USA) |
| CD115-PE Cy7 | BioLegend (CA, USA) |
| CD161-BV711 | BioLegend (CA, USA) |
| F4/80-BV 605 | BioLegend (CA, USA) |
| Zombie Aqua Viability Kit | BioLegend (CA, USA) |
| Ly6C-PerCP | BioLegend (CA, USA) |
| Ly6G-PE Fire 640 | BioLegend (CA, USA) |
| CD11b-APC | Miltenyi Biotech (CA, USA) |

Table S1. Antibody panel with conjugate fluorophore used for tumor flow cytometry.

| **Antibody** | **Company** |
| --- | --- |
| CD45-Pacific blue | BioLegend (CA, USA) |
| CD3-Alexa Fluor 488 | BioLegend (CA, USA) |
| CD4-BV 785 | BioLegend (CA, USA) |
| CD8-APC Fire 750 | BioLegend (CA, USA) |
| CD11c-BV 421 | BioLegend (CA, USA) |
| CD161-BV711 | BioLegend (CA, USA) |
| Zombie Aqua Viability Kit | BioLegend (CA, USA) |
| CD11b-Spark NIR 685 | BioLegend (CA, USA) |
| CD44-BV570 | BioLegend (CA, USA) |
| CD62L-BV650 | BioLegend (CA, USA) |
| CD25-PE Cy7 | BioLegend (CA, USA) |
| IFNγ-PerCP cy5.5 | Invitrogen (CA, USA) |
| Granzyme B- APC | BioLegend (CA, USA) |

Table S2. Antibody panel with conjugate fluorophore used for spleen flow cytometry.
